# Adherence of long-term breast cancer survivors to follow-up care guidelines: a study based on real-world data from the SURBCAN cohort

**DOI:** 10.1007/s10549-022-06563-x

**Published:** 2022-03-15

**Authors:** Paula Santiá, Anna Jansana, Isabel del Cura, Maria Padilla-Ruiz, Laia Domingo, Javier Louro, Mercè Comas, Teresa Sanz, Talita Duarte-Salles, Maximino Redondo, Berta Ibañez, Alexandra Prados-Torres, Xavier Castells, Maria Sala, SURBCAN Group

**Affiliations:** 1 Department of Epidemiology and Evaluation, Hospital del Mar Medical Research Institute (IMIM), Passeig Marítim, 25-29, 08003 Barcelona, Spain; 2 Preventive Medicine and Public Health Teaching Unit (Hospital del Mar – Barcelona Public Health Agency – Pompeu Fabra University), Barcelona, Spain; 3 European Higher Education Area Doctoral Program in Methodology of Biomedical Research and Public Health in Department of Pediatrics, Obstetrics and Gynecology, Preventive Medicine and Public Health, Autonomous University of Barcelona (UAB), Bellaterra, Barcelona Spain; 4 Health Services Research on Chronic Patients Network (REDISSEC), Institute of Health Carlos III, Madrid, Spain; 5 Primary Care Research Unit, Madrid Health Service, Calle San Martín de Porres, 6-5ª planta, 28035 Madrid, Spain; 6 Department of Preventive Medicine and Public Health, Rey Juan Carlos University, Avenida de Atenas, 28922 Alcorcón, Madrid Spain; 7 Research Unit, Costa del Sol Hospital, Biomedical Research Institute of Málaga (IBIMA), University of Málaga, Marbella, Spain; 8 Fundació Institut Universitari per a la recerca a l’Atenció Primària de Salut Jordi Gol i Gurina (IDIAPJGol), Barcelona, Spain; 9 Navarrabiomed, Hospital Complex of Navarra, Public University of Navarra, IdiSNA, Irunlarrea, s/n, 31008 Pamplona, Spain; 10 EpiChron Research Group On Chronic Diseases, Aragon Health Sciences Institute (IACS), IIS Aragon, Miguel Servet University Hospital Pl +2, Paseo Isabel la Católica 1-3, 50009 Saragossa, Spain; 11 Autonomous University of Barcelona (UAB), 08193 Bellaterra, Barcelona Spain; 12 Research Network on Chronicity, Primary Care and Health Promotion (RICAPPS), Institute of Health Carlos III, Madrid, Spain

**Keywords:** Guideline adherence, Long-term survivors, Cancer survivors, Breast neoplasms, Aftercare, Mammography

## Abstract

**Purpose:**

To identify adherence to follow-up recommendations in long-term breast cancer survivors (LTBCS) of the SURBCAN cohort and to identify its determinants, using real-world data.

**Methods:**

We conducted a retrospective study using electronic health records from 2012 to 2016 of women diagnosed with incident breast cancer in Spain between 2000 and 2006 and surviving at least 5 years. Adherence to basic follow-up recommendations, adherence according to risk of recurrence, and overall adherence were calculated based on attendance at medical appointments and imaging surveillance, by year of survivorship. Logistic regression models were fitted to depict the association between adherence and its determinants.

**Results:**

A total of 2079 LTBCS were followed up for a median of 4.97 years. Of them, 23.6% had survived ≥ 10 years at baseline. We estimated that 79.5% of LTBCS were overall adherent to at least one visit and one imaging test. Adherence to recommendations decreased over time and no differences were found according to recurrence risk. Determinants of better overall adherence were diagnosis in middle age (50–69 years old), living in a more-deprived area, having fewer years of survival, receiving primary treatment, and being alive at the end of follow-up.

**Conclusion:**

We identified women apparently not complying with surveillance visits and tests. Special attention should be paid to the youngest and eldest women at diagnosis and to those with longer survival.

## Introduction

Breast cancer is the most frequent cancer in women worldwide. Improvements in diagnosis, treatment, access to health care systems, and screening programs have greatly enhanced the likelihood of surviving the disease [1]. Current data show that up to 85.5% of women with breast cancer will survive 5 years and up to 70%, 10 years after diagnosis [2, 3]. Therefore, the population of long-term breast cancer survivors (LTBCS), defined as women with survival for 5 years or more, is increasing [4]. Although the risks of recurrence and adverse effects are higher in the first few years after diagnosis [5, 6], second breast cancer events can occur at any time and LTBCS continue to experience health problems and disruptions to social life decades after diagnosis. The main concerns include skin disorders, lymphedema, cardiotoxicity, cognitive impairment, bone and musculoskeletal health, pain and neuropathy, premature menopause and infertility, distress, depression, and anxiety, fatigue, sexual health, return to work, and daily activities [7, 8].

The guidelines of the American Society of Clinical Oncology, the European Society of Medical Oncology, and the Spanish Society of Medical Oncology (SEOM) recommend that follow-up include active surveillance of recurrence and new primary tumors, treatment of complications and late adverse effects, management of comorbidities, general preventive care for other conditions as in the rest of the population, and health promotion. All guidelines recommend an annual consultation and mammography for active surveillance [7–11]. Since follow-up by primary care physicians has proven to be safe, efficient, satisfactory, and cost effective [12–14], in Spain, aftercare is shared between primary and hospital care. The SEOM suggests that the frequency and site of appointments among LTBCS should depend on their risk of recurrence. For women considered at low and intermediate risk, one primary care consultation a year is recommended. For those at high recurrence risk, recommendations include consultations every 6 months, shared between primary and hospital care, from the fifth through the 10th year of survival, and once a year in primary care thereafter [11].

There is no established threshold to define adequate adherence and it is usually defined following investigators’ criteria. Adherence to surveillance imaging tests and visits has been assessed separately. Evidence so far indicates that LTBCS visit a physician at least once a year, although not necessarily in primary care; furthermore, they are not screened as recommended, whether because they underuse or overuse recommended tests [15]. Describing the adherence to follow-up recommendations and its determinants is the first step to identify LTBCS with potentially unmet or inadequately addressed needs [8, 16].

In this study, we aimed to (1) estimate adherence to follow-up recommendations in LTBCS in the Spanish SURBCAN cohort, in general and according to recurrence risk and (2) to identify the factors associated with adherence using real-world data.

## Methods

### Setting

The Spanish National Health System (NHS) provides universal coverage and is mainly financed by tax revenue. The system is decentralized and consists of three organizational levels: (1) Central, The Ministry of Health; (2) autonomous communities (AC); and (3) administrative health areas, smallest territorial areas within each AC responsible for the management of the health services offered. Residents are insured under two categories according to their affiliation to the Social Security System: active, for workers contributing to Social Security, and pensioners, for those receiving benefits due to retirement, permanent disability, widow/orphan-hood, or old age. Also, around 16% of the population buys voluntary private health insurance and becomes double covered [17]. Private insurance is unrelated to the public system and care derived from it is not included in public health services databases.

### Study design and population

This observational retrospective study was conducted among the Breast Cancer Survival (SURBCAN) Cohort (ClinicalTrial.gov reference number NCT03846999). Briefly, this cohort includes information on 19,416 women. Of these, 6512 are LTBCS diagnosed with incident cancer between January 1, 2000 and December 31, 2006 in the NHS from five Spanish regions (Andalusia, Aragon, Catalonia, Madrid, and Navarre), aged 18 or older at diagnosis, and who were alive at the beginning of the follow-up period (from January 1, 2012 to December 31, 2016). The remaining 12,904 are women without breast cancer matched two-to-one by age and administrative health area to LTBCS. To be included, women in each group were required to have at least one contact with primary care during follow-up. Information was retrieved during 2018. More information on the SURBCAN cohort is detailed elsewhere [18].

Study participants included LTBCS from three regions of the SURBCAN cohort (Andalusia, Catalonia, and Madrid) that had available information on tumor characteristics and treatment. All participants had survived for between 5 and 12 years at the beginning of follow-up and could be followed up for a maximum of 5 years. Endpoints of the follow-up period were completion of 5 years, death, or being lost to follow-up. We excluded women with less than 6 months of follow-up for any year of survival (*n* = 22).

The study protocol was reviewed and approved by the Ethics Committees of each participating institution. Informed consent was not required.

### Independent variables

Variables were drawn from patients’ routine contacts with primary care and specialized care through electronic health records and from hospital tumor registries.

Socio-demographic variables included country of origin (Spain or other), health coverage (active or pensioner), Medea index (quintiles), and vital status at the end of follow-up (alive or exitus). The Medea index is a deprivation index constructed to measure socioeconomic inequalities [19]. The index is categorized in quintiles and refers to area of residence, with the first quintile being the least-deprived area and the fifth being the most deprived. This information was available at the level of census section for sub-cohorts from Catalonia and Madrid and was missing for Andalusia.

Clinical variables included age at diagnosis (then conflated into < 40, 40–49, 50–59, 60–69, ≥ 70 years old), years of survival at baseline [calculated from date of diagnosis and then grouped in two categories (5–9 years and ≥ 10 years)], tumor behavior at diagnosis (in situ or invasive), treatment received [surgery (yes/no), chemotherapy (yes/no), radiotherapy (yes/no), hormone therapy (yes/no)], and Charlson Comorbidity Index at baseline (0, 1, 2, and ≥ 3). Charlson Comorbidity Index was calculated using the original diagnoses captured by International Classification of Diseases (ICD 9th and 10th edition) or the International Classification of Primary Care (CIAP2), although, excluding breast cancer diagnosis from the algorithm. Risk of recurrence is established by the SEOM according to tumor behavior, tumor receptors, size, invasion, and response to treatment, as well as the genetic platform profile. In the absence of this information for most participants, risk of recurrence was established according to the treatment received: (a) women at low risk, defined as those treated with surgery and radiotherapy only and (b) women at high risk, defined as those treated with chemotherapy either alone or in combination with surgery and/or radiotherapy.

Health services use variables included annual contact rate per women, during the follow-up period. Rates were calculated for primary care (including consultations to any primary care professional and any exam), specialized care (including consultations to any specialized professional and any exam), cancer-related visits (including consultations to primary care physician, gynecologist, medical oncologist, and radio-oncologist), complementary exams (including laboratory, imaging, and other diagnostic tests), imaging tests [including ultrasound, radiology (plain or contrast), magnetic resonance imaging, computed tomography, other imaging test], and mammograms. Type of imaging test was not available for Andalusia; therefore, annual mammogram rate was not calculated for women from the region.

### Outcome variables

The main study variable was adherence to follow-up recommendations. Three types of variables for adherence were created: annual adherence to basic recommendations, annual adherence by risk of recurrence, and overall adherence. Each type was defined twice according to the available information: (1) considering any imaging test (available for the whole cohort, *n* = 2079) and (2) considering mammograms only, when information on type of imaging test was available (*n* = 1758).

Annual adherence to basic recommendations was assessed as follows: women with no visits to a cancer-related physician or with no images per year of survival were considered to have received less than recommended care. If a woman had at least one visit and at least one imaging test or one mammogram, she was considered adherent. If two or more images were mammograms, the woman was considered to have received more than recommended care.

Following SEOM recommendations [11], women at low recurrence risk were considered adherent following the above criteria only if the consultation was performed in primary care. Women at high recurrence risk were considered adherent in year 6th through 10th if they had at least one consultation every 6 months, either in primary or hospital care, and one or more imaging test or one mammogram per year. In year 11th through 17th, women were considered adherent if they had at least one consultation in primary care and one or more imaging tests or one mammogram per year.

Women who adhered to the basic recommendations for more than half their follow-up period were considered overall adherent. When analyzing type of image, we considered women with more than recommended mammograms as adherent.

### Statistical analysis

We performed a descriptive univariate analysis of the cohort using frequencies and percentages for categorical variables, mean, and standard deviation for continuous normally distributed variables and median and inter-quartile range for continuous not normally distributed variables. Health services use is described as annual contact rates per woman. The total number of contacts to each service was used as the numerator and the total number of women-year throughout the study period as the denominator. Percentage of women adherent to basic recommendations and adherent to recommendations according to risk of recurrence is described by survival year (6th through 17th) and trend in adherence was assessed using the *χ*^2^ test for trend. Also, percentage of adherent women on comparison of groups at low and high recurrence risk was assessed yearly using *χ*^2^ test. A descriptive analysis of overall adherence through total follow-up was performed, followed by bivariate analysis between overall adherence and women’s socio-demographic and clinical characteristics, using the chi-square test to assess differences. Logistic regression models were fitted to assess the association between overall adherence and health coverage, Medea index, Comorbidity Index, age at diagnosis, baseline survival, treatment received, and vital status by end of follow-up. Results are presented as adjusted odds ratios (aOR) with its 95% confidence intervals (CI). Statistical tests were two-sided and the significance level was set at *p* < 0.05. All analyses were performed using the statistical software IBM SPSS Statistics 25.

## Results

### Population characteristics

Study participants are described in Table 1. A total of 2079 LTBCS were included. Mean age at diagnosis was 57 years (SD 12.1) and 23.6% of them had survived 10 or more years at the beginning of the study. In all, 55.3% resided in the most-deprived areas and 69.7% of participants had no comorbidity additional to breast cancer. Most (86.7%) were diagnosed with invasive breast cancer. The annual contact rate per woman was 14.4 with primary care and 5.8 with hospital care services. The annual rate of cancer-related visits was 10.6 per woman, most of them being with primary care physicians. Most (86.9%) of the participants survived to the end of the study. Participants were followed up for a median of 4.97 years.

**Table 1:** Participants and health services use

	*N*	%
Participants	2079	100
Country of origin^a^		
Spain	1597	94.78
Other	88	5.22
Health coverage^b^		
Active	550	27.38
Pensioner	1459	72.62
Medea index^c^		
1st quintile	193	11.92
2nd quintile	223	13.77
3rd quintile	308	19.02
4th quintile	521	32.18
5th quintile	374	23.10
Comorbidity index^d^		
0	1428	69.73
1	244	11.91
2	178	8.69
≥ 3	198	9.67
Age at diagnosis (years)		
Mean (SD)	57	(12.09)
Baseline survival (years)		
5 to 9	1589	76.43
10 to 12	490	23.57
Stage at diagnosis^e^		
In situ	274	13.30
Invasive	1786	86.70
Treatment received		
Surgery^f^	1980	95.56
Radiotherapy^g^	1415	68.13
Surgery and radiotherapy only	641	30.92
Chemotherapy^h^	1050	50.53
Hormone therapy^i^	831	39.99
Health services use		
*N* (rate per woman-year)		
Contacts with primary care	141,704	(14.43)
Contacts with specialized care	56,814	(5.79)
Cancer-related visits	103,765	(10.57)
Primary care physician	82,560	(8.41)
Gynecologist	6044	(0.62)
Oncologist^j^	15,161	(1.54)
Complementary exams	38,488	(3.92)
Images	23,067	(2.35)
Mammograms^k^	6090	(0.73)
Final vital status^l^		
Alive	1796	86.93
Exitus	270	13.1
Years of follow-up		
Median (IQR)	4.97	(0.13)
Total	9819.26	

*SD* standard deviation, *IQR* inter-quartile range

Missing values: ^a^394, ^b^70, ^c^460, ^d^31, ^e^19, ^f^7, ^g^2, ^h^1, ^i^1, ^l^13

^d^Adapted from Charlson Comorbidity Index, without breast cancer diagnosis

^j^Includes medical and radio-oncologists

^k^Woman-years of follow-up for mammography rate: 8348.20

### Annual adherence to basic recommendations

When we included all imaging studies, 49.6% (*n* = 1032) of LTBCS were adherent and 3.32% (*n* = 69) consistently received less than recommended care each year of follow-up. The remaining 64.4% switched between adherence and non-adherence during the study period (Table 2). When we analyzed mammograms only, 17.9% (*n* = 315) of women were adherent to basic recommendations each year. When we included women with more than one mammogram in some years of their follow-up, the percentage of adherent women increased to 26.6% (*n* = 468). A total of 19.2% (*n* = 337) received less than recommended care in each year of follow-up and the rest showed irregular adherence over the study period. Assessed annually, the percentage of women adherent to basic recommendations decreased over time. When we analyzed all imaging tests, adherence ranged from 82.8% in the 8th year to 68.1% in the 17th year (*χ*^2^ test for trend, *p* < 0.001); when considering mammography, adherence ranged from 61.9% in the 7th year to 27.5% in the 17th year (*χ*^2^ test for trend, *p* < 0.001). The percentage of women with more than one mammogram per year never exceeded 10%.

**Table 2:** Annual guideline adherence to basic recommendations

Year of survival	Imaging test and consultation adherence		Mammogram and consultation adherence		
	Total	Adherent	Total	Adherent	More than recommended
	*N*	*N* (%)	*N*	*N* (%)	*N* (%)
6th	194	157 (80.93)	167	94 (56.29)	5 (2.99)
7th	560	457 (81.61)	475	294 (61.89)	36 (7.58)
8th	854	707 (82.79)	721	441 (61.17)	55 (7.63)
9th	1132	920 (81.27)	958	578 (60.33)	67 (6.99)
10th	1399	1127 (80.56)	1190	692 (58.15)	82 (6.89)
11th	1410	1084 (76.88)	1195	670 (56.07)	69 (5.77)
12th	1313	998 (76.01)	1114	576 (51.71)	63 (5.66)
13th	1135	856 (75.42)	971	486 (50.05)	56 (5.77)
14th	837	601 (71.80)	713	323 (45.30)	43 (6.03)
15th	556	404 (72.66)	472	210 (44.49)	22 (4.66)
16th	326	229 (70.25)	286	126 (44.06)	9 (3.15)
17th	91	62 (68.13)	80	22 (27.50)	< 5
Total follow-up	2079	1032 (49.64)	1758	315 (17.92)	**–**

### Annual adherence according to recurrence risk

**Fig. 1:**
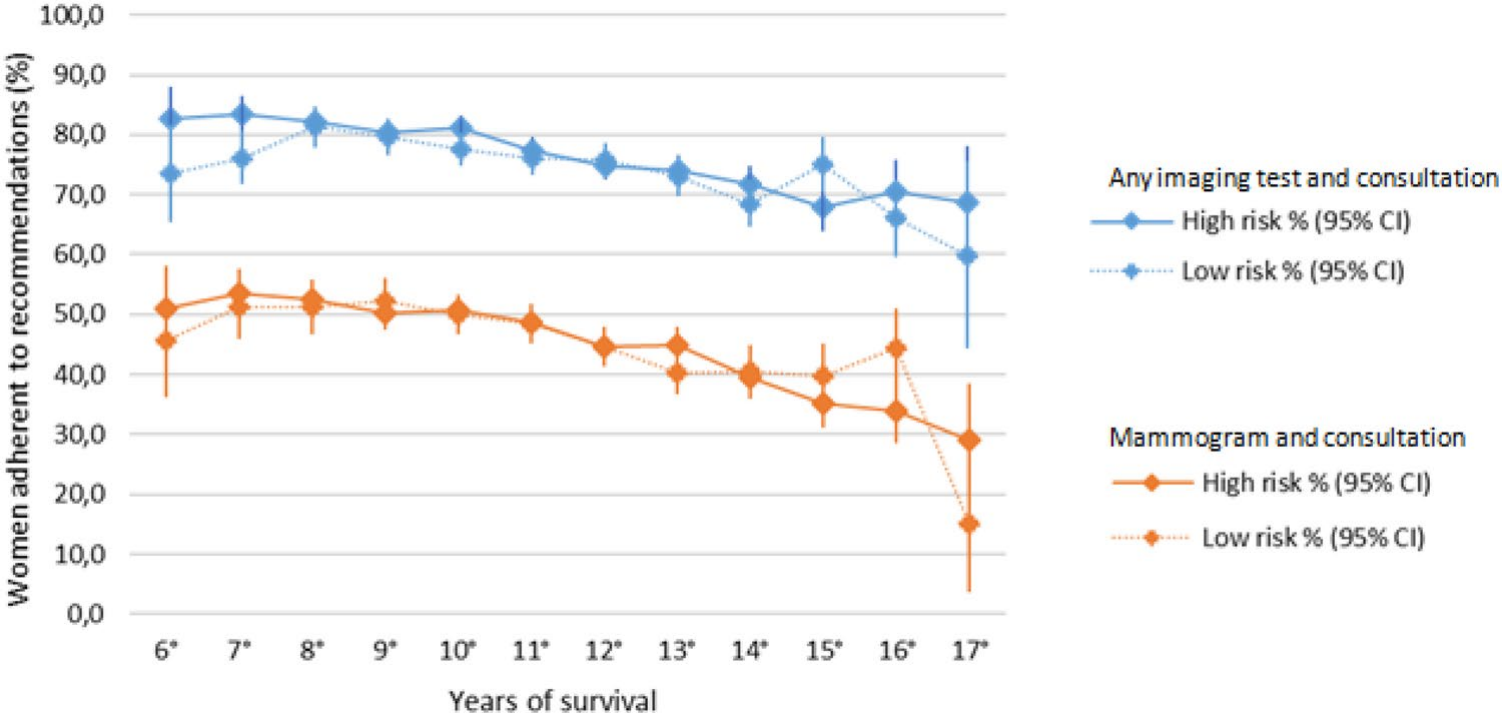
Annual guideline adherence according to risk of recurrence

Figure 1 shows annual adherence by risk of recurrence. In both study groups, the percentage of adherent women decreased over time (*χ*^2^ test for trend, *p* < 0.001). No differences were found in the percentage of adherent women on comparison of groups at low and high recurrence risk in any year (*χ*^2^ test, *p* > 0.05).

### Overall adherence

We estimated that 79.5% of LTBCS were adherent for more than half of the follow-up period to at least one visit and one imaging test. This percentage decreased to 63.3% if only mammograms were included as imaging tests (Table 3). On bivariate analysis, lower adherence was found in women aged 70 years or older, pensioners, those with a higher comorbidity burden, those surviving 10 or more years from diagnosis, and those who had not survived to the end of study period (exitus).

**Table 3:** Participant characteristics according to overall adherence

	Imaging tests and consultation adherence			Mammography and consultation adherence^a^		
	Non-adherent	Adherent	*p*-value	Non-adherent	Adherent	*p*-value
	*N*	*N* (%)		*N*	*N* (%)	
Total	426	1653 (79.5)		646	1112 (63.3)	
Country of origin^b^						
Spain	307	1290 (80.8)	0.112	496	825 (62.5)	0.964
Other	23	65 (73.9)		16	27 (62.8)	
Health coverage^c^						
Active	89	461 (83.8)	**0.008**	148	338 (69.5)	**0.001**
Pensioner	314	1145 (78.5)		494	762 (60.7)	
Medea index^d^						
1st quintile	NA			79	114 (59.1)	**0.001**
2nd quintile	NA			95	128 (57.4)	
3rd quintile	NA			121	187 (60.7)	
4th quintile	NA			154	367 (70.4)	
5th quintile	NA			119	255 (68.2)	
Comorbidity index^e^						
0	272	1156 (81.0)	0.056	399	791 (66.5)	** < 0.001**
≥ 1	141	479 (77.3)		235	308 (56.7)	
Age at diagnosis (years)						
< 40	19	114 (85.7)	** < 0.001**	47	65 (58.0)	** < 0.001**
40–49	82	389 (82.6)		129	266 (67.3)	
50–59	98	487 (83.2)		137	340 (71.3)	
60–69	95	431 (81.9)		153	300 (66.2)	
≥ 70	132	232 (63.7)		180	141 (43.9)	
Baseline survival (years)						
5–9	311	1278 (80.4)	0.062	470	876 (65.1)	**0.004**
≥ 10	115	375 (76.5)		176	236 (57.3)	
Stage at diagnosis^f^						
In situ	55	219 (79.9)	0.906	90	155 (63.3)	0.957
Invasive	364	1422 (79.6)		548	951 (63.4)	
Treatment						
Surgery^g^						
No	25	67 (72.8)	0.095	51	34 (40.0)	** < 0.001**
Yes	396	1584 (80.0)		595	1078 (64.4)	
Radiotherapy^h^						
No	164	498 (75.2)	**0.001**	273	289 (51.4)	** < 0.001**
Yes	261	1154 (81.6)		373	823 (66.8)	
Chemotherapy^i^						
No	244	784 (76.3)	** < 0.001**	344	514 (59.9)	**0.004**
Yes	181	869 (82.8)		302	598 (66.4)	
Hormone therapy^j^						
No	235	1012 (81.2)	**0.026**	443	705 (61.4)	**0.028**
Yes	190	641 (77.1)		203	407 (66.7)	
Final vital status^k^						
Alive	327	1469 (81.8)	** < 0.001**	477	1043 (68.6)	** < 0.001**
Exitus	95	175 (64.8)		162	63 (28.0)	

Medea index was not available for women from Andalusia. In bold, statistically significant *p*-values for *α* = 0.05, calculated using *χ*^2^ test. % Row percentages

*NA* not applicable

^a^321 women from Andalusia excluded for lack of information

Missing values: ^b^Imaging test (I) = 394, Mammogram (M) = 394, ^c^I = 70, M = 16, ^d^M = 139, ^e^I = 31, M = 25, ^f^I = 19, M = 14, ^g^I = 7, ^h^I = 2, ^i^I = 1, ^j^I = 1, ^k^I = 13, M = 13

When all imaging tests were analyzed, adherence was highest among the youngest women (85.7%), while if mammograms only were analyzed, adherence was highest among women aged 50–59 years (71.3%). Analysis by treatment showed that women receiving surgery, radiotherapy and chemotherapy were more adherent than those who did not received these treatments. However, women receiving hormone therapy were less adherent than those not receiving it, when we considered all imaging tests and were more adherent when we considered mammograms alone. Regarding the Medea index, women living in less-deprived areas showed the lowest adherence (59.1% and 57.4%, respectively).

The results of the multivariate logistic regression models are shown in Table 4. When all imaging tests were considered, women aged ≥ 70 years and those who died during follow-up had the lowest odds of being adherent (compared with women aged < 40 years, aOR 0.39, 95% CI 0.21–0.73; compared with surviving women, aOR 0.55, 95% CI 0.40–0.75, respectively). Those receiving radiotherapy and chemotherapy showed higher odds of being adherent (compared with those not receiving these treatments, aOR 1.39, 95% CI 1.09–1.78 and 1.33, 95% CI 1.05–1.70, respectively). Receiving hormone therapy lowered the odds of being adherent by 31% (aOR 0.69, 95% CI 0.54–0.87).

**Table 4:** Determinants of overall adherence in logistic regression analysis

	Imaging tests and consultation adherence			Mammography and consultation adherence^a^		
	Adjusted OR^b^	(95% CI)	*p*-value	Adjusted OR^c^	(95% CI)	*p*-value
Health coverage						
Active	1			1		
Pensioner	0.98	(0.7–1.38)	0.929	0.78	(0.55–1.10)	0.159
Medea index						
1st quintile	NA			1		
2nd quintile	NA			0.83	(0.54–1.27)	0.388
3rd quintile	NA			1.10	(0.73–1.65)	0.648
4th quintile	NA			1.91	(1.30–2.81)	**0.001**
5th quintile	NA			1.66	(1.11–2.49)	**0.013**
Comorbidity index						
0	1			1		
≥ 1	1.10	(0.85–1.43)	0.475	0.85	(0.65–1.10)	0.207
Age at diagnosis (years)						
< 40	1			1		
40–49	0.87	(0.49–1.56)	0.649	1.61	(0.99–2.61)	0.055
50–59	0.95	(0.53–1.72)	0.868	2.45	(1.45–4.15)	**0.001**
60–69	0.85	(0.46–1.56)	0.592	2.07	(1.19–3.61)	**0.010**
≥ 70	0.39	(0.21–0.73)	**0.003**	1.15	(0.64–2.08)	0.644
Baseline survival (years)						
5–9	1			1		
≥ 10	0.81	(0,62–1.05)	0.113	0.73	(0.56–0.94)	**0.017**
Treatment						
Surgery						
No	1			1		
Yes	1.35	(0.8–2.26)	0.262	2.02	(1.20–3.39)	**0.008**
Radiotherapy						
No	1			1		
Yes	1.39	(1.09–1.78)	**0.009**	2.09	(1.64–2.66)	** < 0.001**
Chemotherapy						
No	1			1		
Yes	1.33	(1.05–1.70)	**0.019**	1.29	(1.02–1.63)	**0.036**
Hormone therapy						
No	1			1		
Yes	0.69	(0.54–0.87)	**0.002**	1.31	(1.02–1.69)	**0.035**
Final vital status						
Alive	1			1		
Exitus	0.55	(0.40–0.75)	** < 0.001**	0.18	(0.13–0.27)	** < 0.001**

Medea index was not available for women from Andalusia. In bold, statistically significant *p*-values for *α* = 0.05

*NA* not applicable, *OR* odds ratio

^a^321 women from Andalusia excluded for lack of information

^b^Logistic regression *N* 1963, model predictive probability 80.4%, OR adjusted for all the variables shown

^c^Logistic regression *N* 1572, model predictive probability 71.6%, OR adjusted for all the variables shown

When mammograms were considered, women aged 50 to 69 at diagnosis and those living in deprived areas were more adherent (compared with women aged < 40 years, aOR 2.45, 95% CI 1.45–4.15 for women aged 50 to 59 years, and 2.07, 95% CI 1.19–3.61 for those aged 60 to 69 years; compared with 1st quintile areas, aOR 1.91, 95% CI 1.30–2.81, for those living in 4th quintile areas, and aOR 1.66, 95% CI 1.11–2.49 for those living in 5th quintile areas). In contrast, those who died during follow-up had the lowest odds of being adherent (aOR 0.18, 95% CI 0.13–0.27). Women who had survived 10 or more years from diagnosis at the beginning of follow-up showed 27% lower odds of being adherent than those surviving 5–9 years (aOR 0.73, 95% CI 0.56–0.94). Women receiving any kind of treatment had higher odds of being adherent than those who did not.

## Discussion

Only a few studies have focused on adherence among LTBCSs, including ≥ 10 years survivors [20–23] or in a European context [13, 22–25]. This study shows that a considerable percentage of women (20.5%) in the SURBCAN cohort did not attend an annual consultation or imaging test for half or more of their follow-up. Non-adherence was even higher when imaging was limited to mammograms.

This study is the first to analyze adherence with real-world data among Spanish LTBCS, and the results are in line with those of studies performed in other contexts [21–23, 26–31].

Irrespective of imaging type and recurrence risk, adherence decreased over time. This pattern has been reported by other studies [22, 27, 32, 33]. This may be explained by a decreasing concern about recurrence over the years or tailored schedules in which the estimated risk of recurrence influences the choice to follow guideline recommendations [34]. However, we found no differences in adherence according to risk of recurrence. This finding may be due to a simplified definition of risk or to low statistical power.

Age at diagnosis seems to play an important role in adherence. Compared with women under 40 years, those aged ≥ 70 years at diagnosis were less likely to be adherent when we analyzed all imaging tests, whereas women aged between 40 and 69 years were more likely to be adherent when we analyzed mammograms only. This result aligns with previous studies [21–23, 27]. A possible explanation may be that younger women undergo surveillance images other than mammograms due to denser breast tissue and that older women are less concerned about second primary tumors or recurrences. In elderly women, a possible explanation may be competing medical needs, which may not be included in comorbidity indexes, a reduction in perceived benefit from surveillance, or unclear guideline recommendations for older survivors [31, 32, 35, 36].

Prior studies have reported that women with a high comorbidity burden were less likely to receive surveillance mammograms [15, 20, 21, 23, 28], a result which we were unable to confirm. This could be because we used a static variable assessed at the beginning of the follow-up period or because we excluded breast cancer diagnosis in the Charlson Comorbidity Index. However, women not surviving to the end of the study were indeed less adherent. This may be explained by the “sick stopper effect” whereby patients who become increasingly ill forego preventive care [37, 38]. Conversely, our results could also be explained by the fact that non-adherence to guidelines may decrease survival rates [39]. However, without information on causes of death, recurrences, or metastasis we were unable to prove these hypotheses.

Similar to our findings, other studies have reported that treatment with radiation and chemotherapy was associated with higher rates of adherence [21, 23–25, 32–34]. Ruddy et al. [31] found that woman treated with chemotherapy were less likely to undergo mammography, although this association was weaker in the fifth year of follow-up than in the first and was not assessed beyond 5 years. The association between hormone therapy and adherence is less clear [18, 26]. Our results suggest that receiving hormone therapy decreases adherence when all kinds of imaging tests were considered, but increases adherence when considering mammograms only. A possible explanation may be that LTBCS with worse prognosis do not receive hormone therapy as part of treatment. These women may follow different recommendations and receive more visits and undergo imaging tests other than mammography.

Unlike other authors [22], we found that women living in deprived areas were more likely to be adherent. Our results may be biased by consultations and imaging tests performed at private clinics, which could not be ascertained. Women living in lower-quintile areas are more likely to have double-health coverage and may be adequately followed up in the private sector and not captured in our public health services databases [17, 40].

### Strengths and limitations

In our study, we had access to complete longitudinal data on health services use for up to 5 years, for a large population of insured LTBCS in Spain. Other strengths include a mixed age population and a novel focus on long-term survival using real-world data. Although we consider regional diversity a strength of this study and all three AC work within the framework of the Spanish National Health Service, each region has a different health service organization and management model. Therefore, the availability of data differs in each sub-cohort. Electronic health records from primary care and hospital databases provide detailed information on demographics, breast cancer characteristics, treatment, consultations, and comorbidities, reducing memory bias and allowing chronic processes to be analyzed from a multi-causal approach. This study provides valuable information to improve current practices and survivors’ adherence to follow-up recommendations.

This study also has some limitations. Adherence could be overestimated. Because we could not ascertain the reasons for consultations and test prescriptions or symptoms at the time of the test, we could have included visits unrelated to breast cancer follow-up and diagnostic exams. While relevant, no data were available on the type and site of surgery, recurrence, metastasis, or menopausal status. In addition, it is difficult to ascertain time intervals, because some women may receive two mammograms in a year (at the beginning and at the end) and therefore none the following year. We intended to minimize misclassification with overall adherence analysis. Most of our population was Spanish (94.4%) with no other comorbidities (69.7%) at baseline. Estimates may be lower in populations with less access to medical services and those with more comorbid conditions [32, 37]. Data were drawn from the electronic health records from the Spanish National Health Service and do not include private sector contacts. However, use of private sector for oncology processes is low in Spain [41]. The association between socioeconomic status and adherence was addressed using an ecological measure of deprivation, rather than an individual one, which may lead to bias. Moreover, the deprivation index used in this study was based on national 2001 census data.

Other aspects that might influence adherence and were not considered in this study include participants’ educational level, preferences, fear and health literacy, and provider recommendations. These may act as unobserved confounders.

## Conclusion

A considerable percentage of women in contact with health services are not been properly followed up. These findings reinforce the need for active engagement of all breast cancer survivors in long-term aftercare, especially the youngest and eldest at diagnosis and those with the longest survival. Further research is needed to better understand patient and provider attitudes to survivorship care as well as guideline adequacy.

## Data Availability

The datasets generated and/or analyzed during the current study are available from the corresponding author on reasonable request. The original cohort is available at BMJ Open, https://doi.org/10.1136/bmjopen-2020-040253.
